# Combined Expression of FOXO and Tribbles Proteins Predicts Survival of Glioma Patients

**DOI:** 10.32604/or.2026.083560

**Published:** 2026-10-28

**Authors:** Bruno F. Santos, Ana-Teresa Maia, Inês Grenho, André Besouro-Duarte, Juan M. Sepúlveda-Sánchez, Bibiana I. Ferreira, Wolfgang Link

**Affiliations:** 1 Algarve Biomedical Center Research Institute—ABC-RI, Universidade do Algarve, Campus de Gambelas, Faro, Portugal; 2 Algarve Biomedical Center (ABC), Campus de Gambelas, Faro, Portugal; 3 Unidade Local de Saúde do Algarve (ULSAlg), Rua Leão Penedo, Faro, Portugal; 4 Faculdade de Medicina e Ciências Biomédicas, Universidade do Algarve, Campus de Gambelas, Faro, Portugal; 5 RISE-Health, University of Algarve, Campus de Gambelas, Faro, Portugal; 6 Neuro-Oncology Unit, Hospital HM Sanchinarro (CUHMED), Instituto de Investigación 12 de Octubre (I+12), Madrid, Spain; 7 Sols-Morreale Biomedical Research Institute (IIBM), Spanish National Research Council (CSIC), Universidad Autónoma de Madrid (UAM), Madrid, Spain

**Keywords:** FOXO, tribbles, glioblastoma, prognostic biomarker, patient stratification, therapeutic target

## Abstract

**Background:** High-grade gliomas remain therapeutically challenging and are associated with poor survival outcomes. Current biomarkers inadequately stratify patients for therapy selection and fail to support early detection of disease progression. The FOXO transcription factors and Tribbles pseudokinases are key regulators of the PI3K/AKT pathway and have been implicated in cancer progression, however, their prognostic value in gliomas remains unclear. This study aimed to determine whether transcriptional profiles of FOXO and Tribbles family members predict survival in glioma patients. **Methods:** Using RNA-seq data from TCGA and CGGA, along with microarray datasets REMBRANDT and Gravendeel, we analyzed the association of FOXO1, FOXO3, FOXO4, FOXO6 and TRIB1, TRIB2, TRIB3 mRNA levels with overall survival and WHO classification. We also experimentally validated the functional role of FOXO1 and TRIB3 in glioblastoma cells through gene depletion assays. **Results:** Using the TCGA RNA-seq cohort, we identified a FOXO/Tribbles transcriptional signature characterized by ↑FOXO1, ↓FOXO3/4 and ↑TRIB1/2/3, which was subsequently evaluated in independent microarray cohorts that confirmed the prognostic relevance of coordinated FOXO/Tribbles family dysregulation despite cohort-specific differences in the optimal gene combinations. Grade 4 tumors exhibited elevated FOXO1 and TRIB1/TRIB2/TRIB3, but reduced FOXO3/FOXO4 levels compared to lower-grade gliomas. Experimental validation supported a potential role of FOXO1 and TRIB3 in promoting glioblastoma cell viability. **Conclusion:** The combined FOXO/Tribbles expression signature (↑FOXO1/↓FOXO3/FOXO4/↑TRIB1/TRIB2/TRIB3) may represent a candidate prognostic biomarker requiring further validation before clinical application, particularly involving emerging agents directed against TRIB2, TRIB3, and FOXO1, as well as small-molecule activators of FOXO3.

## 1 Introduction

Glioblastoma (GB) is the most common and aggressive primary brain tumor in adults, accounting for almost half of the primary malignant brain tumors. In 2021, the World Health Organization (WHO) updated its classification of the central nervous system (CNS) tumors (WHO CNS 5th edition classification, WHO CNS5) to incorporate molecular data, simplifying the system for improved diagnostic accuracy and prognostic assessment [1]. GB treatment includes surgical resection followed by radiation therapy and Temozolomide (TMZ) chemotherapy. Nonetheless, GB remains an incurable disease with a median survival of only 15 months due to therapy resistance and recurrence [2]. Therefore, there is an urgent need for better treatments that can overcome drug resistance, along with biomarkers to stratify risk or guide therapy.

Several studies have demonstrated a prominent role of the PI3K/AKT signaling pathway in glioma development and progression, with alterations in this pathway observed in approximately 90% of GB [1,3]. FOXO transcription factors have been characterized as major transcriptional effectors of the PI3K/AKT pathway [4] and the Tribbles homologue 2 (TRIB2) was identified as a member of the Tribbles family of pseudokinases as a potent repressor of FOXO proteins [5]. Here we explore for the first time the combined role of FOXO and Tribbles proteins in driving glioma aggressiveness. FOXO proteins, a family of transcription factors including FOXO1, FOXO3, FOXO4 and FOXO6 [6], play a key role in regulating the expression of genes involved in cell proliferation, metabolism, apoptosis, autophagy, and stress resistance [7] and are often inactivated in human cancers [8]. Additionally, variants of FOXO3 have been associated with extreme human longevity [8]. A breakthrough in the functional characterization of FOXO transcription factors was achieved by the generation of triple knockout mice lacking FOXO1, 3 and 4, that established FOXO as *bona fide* tumor suppressors [9]. Conversely, an oncogenic role of FOXOs in tumor progression, metastasis, and therapy resistance has emerged [10]. These proteins are regulated by reversible post-translational modifications that convert external stimuli into specific transcriptional programs [11]. Under stress conditions or in the absence of growth factors, FOXOs are active in the nucleus. Yet, in the presence of growth factors, or in many cancer cells where the PI3K/AKT pathway is constitutively active, all FOXO isoforms except FOXO6 are exported to the cytoplasm where they remain inactive [8]. Thus, the pharmacological modulation of FOXO is considered an attractive therapeutic approach for treating cancer and age-related diseases [12]. The extensive functional diversity of these proteins suggests that the benefit of FOXO activation depends on the isoform and cellular context. While the different FOXO isoforms bind to the same consensus DNA sites in target gene promoters, it is increasingly evident that they also exert non-redundant functions in specific tissues [6]. The Tribbles protein family in mammals consists of three isoforms, TRIB1, TRIB2 and TRIB3. These proteins contain a conserved kinase-like domain. However, instead of phosphorylating substrates, these proteins function as protein scaffolds that integrate and modulate key signaling pathways involved in proliferation, survival, and differentiation [13]. TRIB2 has been established as an oncogene in melanoma and high levels of this protein confer resistance to several drugs used for cancer treatment [5,14,15]. While TRIB2 has been implicated in cancer progression and therapy resistance across numerous cancer types [16], and TRIB1 is also considered as an oncogene in various malignancies, the role of TRIB3 is more complex and context-dependent. Studies indicate that the contribution of TRIB3 to cancer progression varies, potentially acting as either a tumor suppressor or an oncogene depending on the specific cellular environment [17,18,19,20,21]. However, the clinical relevance of these observations has yet to be fully established.

The specific objective of this study is to investigate the association between FOXO/Tribbles expression patterns and clinical outcomes in glioma patients, to determine the predictive value of specific FOXO/Tribbles signatures for prognosis—particularly whether high FOXO1 and low FOXO3/4 together with high TRIB1/2/3 levels correlate with worse outcomes—and to assess whether such signatures can identify glioma patients who may benefit from novel therapeutics targeting these factors.

## 2 Material and Methods

### 2.1 Datasets

#### 2.1.1 RNA-Seq Datasets

RNA-seq data was obtained from two independent databases, The Cancer Genome Atlas (TCGA, https://portal.gdc.cancer.gov/) [22] and The Chinese Glioma Genome Atlas (CGGA, http://www.cgga.org.cn/) [23].

Two independent datasets, The Cancer Genome Atlas Glioblastoma Multiforme (TCGA-GBM) and The Cancer Genome Atlas Low Grade Glioma (TCGA-LGG), comprising a total of 705 samples, were directly downloaded from the TCGA database into RStudio (version 2023.3.0.386) in September 2024 using the TCGAbiolinks (version 2.32.0) R package [24]. TCGA data was reclassified according to the WHO CNS5 standards [25]. The reclassified dataset comprises 113 grade 2 astrocytomas, 98 grade 3 astrocytomas, 24 grade 4 astrocytomas, 81 grade 2 oligodendrogliomas, 70 grade 3 oligodendrogliomas, 221 GB and 98 unclassified samples.

Data from the CGGA database (1018 samples) was obtained from two independent datasets (mRNAseq_693 and mRNAseq_325), downloaded from the CGGA portal in September 2024 and uploaded to RStudio. These datasets comprise 175 grade 2 astrocytomas, 214 grade 3 astrocytomas, 112 grade 2 oligodendrogliomas, 9 grade 3 oligodendrogliomas, 94 grade 2 mixed type, 21 grade 3 mixed type, 388 GB and 5 unclassified samples.

For both TCGA and CGGA datasets, read counts (STAR-counts) were converted to transcript per million (TPM) to reduce technical variability between datasets. Table S1 provides an overview of the demographic characteristics and disease features for both RNA-seq datasets.

#### 2.1.2 Microarray Datasets

REMBRANDT (GSE68848) [26] and the Gravendeel (GSE16011) [27] microarray cohorts (Affymetrix HG-U1333Plus2) were available from Gene Expression Omnibus (GEO, https://www.ncbi.nlm.nih.gov/geo/query/acc.cgi). Both databases were downloaded in September 2024 and uploaded into RStudio using GEOquery (version 2.81.21) [28] and Biobase (version 2.64.0) [29] R packages. Specifically, the pre-processed, log-transformed Series Matrix files containing Robust Multi-array Average (RMA) normalized expression values were directly imported into RStudio. In cases where multiple probes were used for one gene, their average expression levels were used. Matching clinical data for REMBRANDT and Gravendeel datasets was obtained through GEO or GlioVis (https://gliovis.bioinfo.cnio.es/), respectively. Rembrandt dataset comprises 61 grade 2 astrocytomas, 53 grade 3 astrocytomas, 27 grade 2 oligodendrogliomas, 22 grade 3 oligodendrogliomas, 4 grade 2 mixed type, 3 grade 3 mixed type, 190 GB and 192 unclassified samples. The Gravendeel dataset comprises 13 grade 2 astrocytomas, 16 grade 3 astrocytomas, 8 grade 2 oligodendrogliomas, 44 grade 3 oligodendrogliomas, 3 grade 2 mixed type, 25 grade 3 mixed type and 159 GB. Table S1 provides an overview of the demographic characteristics and disease features for both microarray datasets.

### 2.2 Ethics Statement

This study involved only the analysis of publicly available, de-identified human gene expression and clinical data from The Cancer Genome Atlas (TCGA), the Chinese Glioma Genome Atlas (CGGA), the REMBRANDT dataset (GSE68848), and the Gravendeel dataset (GSE16011). As such, no institutional review board approval or informed consent was required. All data used is compliant with relevant ethical guidelines and regulations.

### 2.3 Cell Culture and Maintenance

Human glioblastoma cell lines U-87 MG (HTB-14) and U-118 MG (HTB-15) were obtained from the American Type Culture Collection (ATCC; Manassas, VA, USA). All cell lines were authenticated by short tandem repeat (STR) profiling. U-87 MG and U-118 MG cells were cultured in Dulbecco’s Modified Eagle Medium (DMEM) (Corning, 10-013-CV, New York, NY, USA) supplemented with 10% fetal bovine serum (Cytiva, SV30160.03, Marlborough, MA, USA) and 1% penicillin-streptomycin (PanReac AppliChem, A8943, Darmstadt, Germany). Cell cultures were maintained in a humidified incubator at 37°C with 5% CO_2_ and passaged when confluent using 1X trypsin-EDTA (ChemCruz, sc-391060A, Santa Cruz Biotechnology, Dallas, TX, USA). All experiments were performed using mycoplasma-free cells during their logarithmic growth phase.

### 2.4 FOXO1 and TRIB3 Depletion

FOXO1 knockdown was achieved using small interfering RNA (siRNA), whereas TRIB3 knockout was generated via a CRISPR/Cas9-mediated approach. U87 glioblastoma cells were transfected with siRNA targeting FOXO1 (25 nM; Dharmacon, L-003006-00-0005, Lafayette, CO, USA) or a non-targeting scramble control (25 nM; Dharmacon, LD-001810-01-20) using DharmaFECT siRNA Transfection Reagent (Dharmacon, T-2001-03) according to the manufacturer’s protocol. For TRIB3 depletion, U118 glioblastoma cells were edited using the CRISPR/Cas9 system. Two independent guide RNAs (gRNAs) with the sequences 5′-GCCCACTTCGAGCTCGTTTC-3′ and 5′-GTTGCACGATCTGGAGCAGT-3′ were individually cloned into a Cas9 expression vector (Plasmid #62988; Addgene) and delivered into cells using Lipofectamine 2000 (Thermo Fisher Scientific, 11668019, Waltham, MA, USA). Following selection with puromycin (1 μg/mL; Alfa Aesar, J61278, Ward Hill, MA, USA), single-cell clones were isolated by limiting dilution and expanded. TRIB3 knockout was confirmed by immunoblotting.

### 2.5 Viability Assay

Cell viability was assessed using the MTT assay. Briefly, cells were seeded in 96-well plates at a density of 5 × 10^3^ cells per well in complete growth medium (DMEM, 10% FBS, 1% Pen/Strep), with wells containing only medium serving as blanks. After 72 h, the medium was replaced with MTT solution (0.5 mg/mL; Alfa Aesar, L11939, Tewksbury, MA, USA), and cells were incubated for 3 h at 37°C. The resulting formazan crystals were dissolved in 100 μL of DMSO, and absorbance was measured at 570 nm and 650 nm using a microplate reader (SpectraMax iD3, Molecular Devices, San Jose, CA, USA). The 650 nm readings were subtracted from the corresponding 570 nm readings to correct for non-specific background, and blank values were subsequently subtracted from all sample readings. Cell viability was expressed as fold change relative to U118 WT cells or scramble transfected U87 cells. All experiments were carried out in triplicate and repeated in at least three independent experiments (n = 3).

### 2.6 Western Blot

Cells were lysed in CST buffer containing 20 mM Tris-HCl (pH 7.5) (Fisher Bioreagents, BP152, Waltham, MA, USA), 150 mM NaCl (Merck, 1.06404.5000, Darmstadt, Germany), 1% Triton X-100 (Amresco, M143, Solon, OH, USA), 50 mM NaF (VWR, 27858.290, Radnor, PA, USA), 1 mM ethylenediaminetetraacetic acid (EDTA; Sigma-Aldrich, E5134, St. Louis, MO, USA), 1 mM aminopolycarboxylic acid (EGTA; PanReac AppliChem, A0878), 2.5 mM pyrophosphate (Santa Cruz Biotechnology, Dallas, TX, USA), 2 mM β-glycerophosphate (Santa Cruz Biotechnology), 1 mM sodium orthovanadate (Na3VO4; Sigma-Aldrich), 100 nM calyculin A (ChemCruz, sc-24000), and 1x protease inhibitors cocktail (Thermo Fisher Scientific, A32955).

Protein concentrations were determined using the Bradford reagent (ThermoScientic, Thermo Fisher Scientific, J61522.K2). Equal amounts of protein (40 μg per lane) were separated by 10% SDS-PAGE and transferred onto PVDF membranes. Membranes were blocked with 5% (w/v) non-fat milk in Tris-buffered saline containing 0.1% Tween 20 (TBS-T) for 1 h at room temperature with agitation. After blocking, membranes were incubated overnight at 4°C with primary antibodies against TRIB3 (1:1000; Cat# ab75846; Abcam, Cambridge, UK) and FOXO1 (1:500; Cat# 2880; Cell Signaling Technology, Danvers, MA, USA). Membranes were washed three times for 5 min with TBS-T and incubated for 1 h at room temperature with an HRP-conjugated anti-rabbit secondary antibody (1:10,000; Cat# NA934; Amersham, Marlborough, MA, USA) or HRP-conjugated anti-mouse secondary antibody (1:10,000; Cytiva, NA931, Marlborough, MA, USA). Bands were visualized using enhanced chemiluminescence (ECL) on iBright 1500 imaging system (Invitrogen, Thermo Fisher Scientific, Waltham, MA, USA). Blot images were processed in Fiji/ImageJ (version 1.53f51) by cropping the regions of interest without altering image content and assembled into figures using Microsoft PowerPoint (version 2606). GAPDH (1:1000; Cat# sc-25778; Santa Cruz Biotechnology, Dallas, TX, USA) or α-Tubulin (1:1000; Cat# T9026; Sigma-Aldrich, St. Louis, MO, USA) served as loading controls. 

### 2.7 Statistical Analysis

All statistical analyses related to the bioinformatic evaluation of glioma datasets were performed in R (version 4.4.1) using RStudio (version 2023.3.0.386). Survival analysis was performed with Kaplan-Meier, using the survival (version 3.7.0) [30] and survminer (version 0.4.9) [31] R packages. The primary endpoint was death from any cause, with surviving subjects censored at the date of their last known contact. Kaplan–Meier survival curves were compared using the log-rank test. Gene expression cutoffs were defined using quartiles for single-gene analyses to maximize the contrast between low- and high-expression groups. Unless otherwise specified, all statistical tests were two-sided and *p* values < 0.05 were considered statistically significant. To account for multiple testing across the seven predefined genes analyzed, Bonferroni correction was applied by multiplying the *p* values by seven. The correction did not alter the statistical significance of any reported findings. For combinatorial analyses, median-based cutoffs were used to avoid generating excessively small or empty subgroups that could compromise statistical robustness. The Concordance Index (C-index) was calculated using the concordance function from survival (version 3.7.0) R package [30] to assess the association between the prognostic variable under investigation and patient survival outcomes. The C-index quantifies the agreement between the ranking of patients according to the prognostic variable and the observed survival times. Expression levels of FOXO and Tribbles genes in glioblastoma and other glioma types were visualized as log2-transformed TPM values using ggplot2 (version 3.5.1) [32]. Differences between groups were assessed using two-sided Student’s *t*-tests. This analysis was restricted to the TCGA cohort, as it was the only dataset with diagnoses updated according to the WHO 2021 classification, ensuring consistent and standardized tumor classification across samples. Multivariate Cox proportional hazards regression was performed in the TCGA cohort using the survival (version 3.7.0) [30] R package to assess the independent prognostic value of the combined transcriptional profiles of FOXO and Tribbles on the overall survival. This analysis was restricted to TCGA because it was the only dataset with complete clinical and molecular annotations and diagnoses updated according to the WHO 2021 classification. Hazard ratios (HRs) and 95% confidence intervals (CIs) were estimated by fitting the Cox model while adjusting for age (stratified into young, middle-aged, and elderly [33]), gender, IDH mutation status, 1p/19q codeletion, MGMT promoter methylation, and WHO classification.

Statistical analyses of cell viability experiments were performed using GraphPad Prism (version 9.5.1). Data is presented as the mean ± standard error of the mean (SEM) from three independent biological experiments (n = 3), each performed using triplicate technical replicates. Comparisons between two groups were performed using two-sided unpaired Student’s *t*-tests, whereas comparisons involving more than two groups were analyzed using one-way analysis of variance (ANOVA) followed by Dunnett’s test for multiple comparisons. Parametric statistical tests were performed under the assumption of approximate normality.

## 3 Results

### 3.1 High FOXO1, Low FOXO3/FOXO4, and Elevated TRIB1/2/3 mRNA Levels Form a Molecular Signature Predictive of Clinical Outcomes

To investigate if mRNA levels of FOXO family members were associated with the prognosis of glioma patients, we analyzed the mRNA levels of a total of 705 samples from the TCGA database, using RStudio. Quartiles were established as the cut-off to define low expression levels under the first quartile and high levels above the third quartile. Kaplan-Meier curves show that the patient survival rate is dependent on FOXO mRNA levels. High levels of FOXO1 (*p* < 0.0001, Fig. 1A) and low levels of FOXO3 (*p* < 0.0001, Fig. 1B), FOXO4 (*p* < 0.0001, Fig. 1C) or FOXO6 (*p* = 0.027, Fig. 1D) are significantly associated with a worse prognosis. Interestingly, FOXO6 mRNA levels have a considerably lower capacity to separate patients according to survival than the rest of FOXOs (Fig. 1D). Given that FOXO expression is tissue-specific, and their functions can overlap, with one isoform potentially compensating for another, we assessed whether the combined expression of various FOXO isoforms was associated with patient prognosis. We observed that patients with poorer survival rates share the characteristic of having high mRNA levels of FOXO1, along with low levels of FOXO3 and FOXO4, irrespective of their FOXO6 levels (Fig. S1A). We validated our findings using an independent dataset of glioma RNA-seq data (CGGA), comprising 1018 samples, and found that high expression of FOXO1 (*p* < 0.0001, Fig. S1B) or low expression of FOXO4 (*p* < 0.0001, Fig. S1C) was significantly associated with poorer patient survival. In contrast, transcript levels of FOXO3 (*p* = 0.25, Fig. S1D) and FOXO6 (*p* = 0.45, Fig. S1E) did not show a significant correlation with patient survival.

Next, we sought to investigate if Tribbles transcript levels would also be associated with different survival rates. We found that high mRNA levels of TRIB1 (*p* < 0.0001, Fig. 1E), TRIB2 (*p* < 0.0001, Fig. 1F) or TRIB3 (*p* < 0.0001, Fig. 1G) were significantly associated with worse patient prognosis. Moreover, patients who expressed combined high levels of all three Tribbles members were associated with the worst survival rates (Fig. S1F). Importantly, elevated mRNA expression of TRIB1 (*p* < 0.0001, Fig. S1G), TRIB2 (*p* < 0.0001, Fig. S1H), or TRIB3 (*p* < 0.0001, Fig. S1I) in CGGA patients was associated with significantly worse survival, aligning with our results from the TCGA dataset.

FOXO transcription factors are downstream effectors of the PI3K/AKT pathway whose activity is modulated by TRIB2 and/or TRIB3 levels in many cancers [13]. Therefore, we sought to investigate if combined expression of these proteins would have a major impact on prognosis in gliomas. Our analysis revealed that patients who simultaneously expressed high levels of FOXO1 and low levels of FOXO3/4 plus high levels of TRIB1/2/3 represent the worst prognosis group of gliomas patients, for both TCGA (*p* < 0.0001, Fig. 1H) and CGGA (*p* < 0.0001, Fig. S1J) datasets. These findings suggest a potential functional relationship between FOXO isoforms and Tribbles family proteins in promoting glioma progression and highlight this specific molecular signature as a potential prognostic indicator for poor clinical outcomes in glioma patients. In order to implement these findings in the clinic they require further experimental validation.

**Figure 1:**
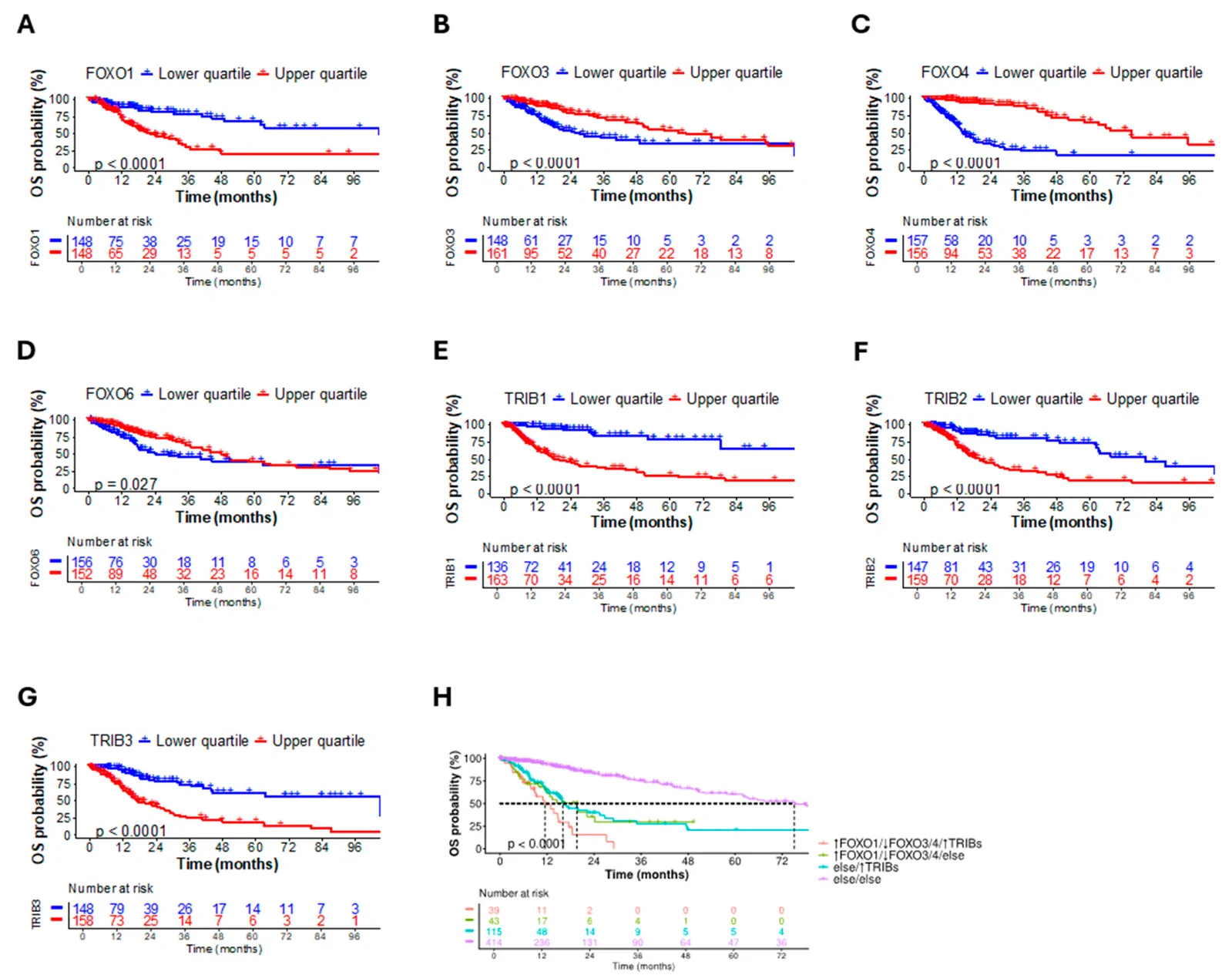
High FOXO1, low FOXO3/FOXO4, and elevated TRIB1/2/3 mRNA levels form a molecular signature predictive of clinical outcomes. (**A**–**G**) Kaplan-Meier plots of FOXO and Tribbles for TCGA glioma samples. The lower quartile, in blue, and the upper quartile, in red, were established as the cut-off for low and high expression levels, respectively. (**A**) FOXO1; (**B**) FOXO3; (**C**) FOXO4; (**D**) FOXO6; (**E**) TRIB1; (**F**) TRIB2; (**G**) TRIB3. (**H**) Kaplan-Meier plot of a combination of high levels of FOXO1 and low levels of FOXO3/4 plus high levels of Tribbles for TCGA glioma samples. The median value was set as the cut-off. “Else” means every other possible combination of expressions than the stated in comparison. High FOXO1 and low FOXO3/4 mRNA levels combined with high levels of TRIB1/2/3 (↑FOXO1/↓FOXO3/4/↑TRIBs) are represented in pink. All *p*-values refer to log-rank test. Abbreviations: OS, overall survival; FOXO, Forkhead box O; FOXO1, Forkhead box O1; FOXO3, Forkhead box O3; FOXO4, Forkhead box O4; FOXO6, Forkhead box O6; TRIB1, Tribbles homolog 1; TRIB2, Tribbles homolog 2; TRIB3, Tribbles homolog 3; TRIBs, Tribbles; mRNA, messenger RNA.

### 3.2 Independent Glioma Datasets Validate the mRNA Signature and Its Robustness

To further validate our findings, we analyzed two independent microarray datasets: REMBRANDT (GSE68848) and Gravendeel (GSE16011). In the REMBRANDT dataset, which includes 552 glioma samples, the expression patterns of FOXO family members showed to be consistent with the predictive signature described above. Specifically, elevated FOXO1 expression levels (*p* = 0.032, Fig. 2A) and reduced levels of FOXO3 (*p* < 0.0001, Fig. 2B), FOXO4 (*p* = 0.00065, Fig. 2C), and FOXO6 (*p* = 0.00042, Fig. 2D) were significantly associated with poorer patient prognosis. Regarding the Tribbles family members, no significant association was found between TRIB1 transcript levels and patient survival (*p* = 0.25, Fig. 2E). However, high expression levels of TRIB2 (*p* = 0.0091, Fig. 2F) and TRIB3 (*p* < 0.0001, Fig. 2G) correlated with worse survival rates. Among all possible combinations of FOXO and Tribbles family members evaluated, the expression profile associated with the poorest survival in the REMBRANDT cohort consisted of low FOXO1/3/4/6 together with high TRIB1/2/3 expression (↓FOXOs/↑TRIBs) (*p* < 0.0001, Fig. 2H). This differs from the direction observed for FOXO1 alone, illustrating that the prognostic effect of individual genes is not necessarily preserved when evaluated as part of a multigene transcriptional profile.

In the Gravendeel microarray dataset, which includes 268 glioma samples, the association between expression levels and survival was consistent with our findings from the TCGA dataset. Specifically, low levels of FOXO3 (*p* < 0.0001, Fig. 2I) and FOXO4 (*p* < 0.0001, Fig. 2J), along with high levels of FOXO1 (*p* < 0.0001, Fig. 2K), TRIB1 (*p* < 0.0001, Fig. 2L), TRIB2 (*p* = 0.00035, Fig. 2M) and TRIB3 (*p* = 0.0024, Fig. 2N) are significantly correlated with poorer prognosis. Notably, this dataset did not include expression levels for FOXO6. For these samples, among all combinations evaluated, the profile with the strongest association with poor survival consisted of high levels of FOXO1 and low levels of FOXO4 plus high levels of TRIB1/3 and low levels of TRIB2 (↑FOXO1/↓FOXO4/↑TRIB1/3/↓TRIB2) (*p* < 0.0001, Fig. 2O). This differs from the individual association observed for TRIB2, highlighting that the combined transcriptional profile may reflect interactions among FOXO and Tribbles family members rather than additive effects of individual genes.

Taken together, these findings show that, despite some variability across different populations, patients with low FOXO3, FOXO4 and FOXO6, and with high FOXO1, TRIB1, TRIB2 and TRIB3 mRNA levels have significantly worse survival rates. The combined analyses evaluate the net effect of multiple FOXO and Tribbles family members rather than the prognostic contribution of each gene individually. Consequently, the direction of association observed for an individual gene is not necessarily preserved within the multigene expression profile. Because TCGA represents the largest and most comprehensively annotated RNA-seq cohort, the FOXO/Tribbles signature derived from this dataset constitutes the primary signature proposed in this study, whereas the REMBRANDT and Gravendeel datasets provide independent validation supporting the prognostic relevance of coordinated FOXO/Tribbles dysregulation. These discrepancies suggest that further studies are needed to fully establish their relationship with glioma patient survival. Importantly, the data suggests that combined transcriptional profiles of FOXO and Tribbles family members could be used to predict the clinical outcome of gliomas patients.

**Figure 2:**
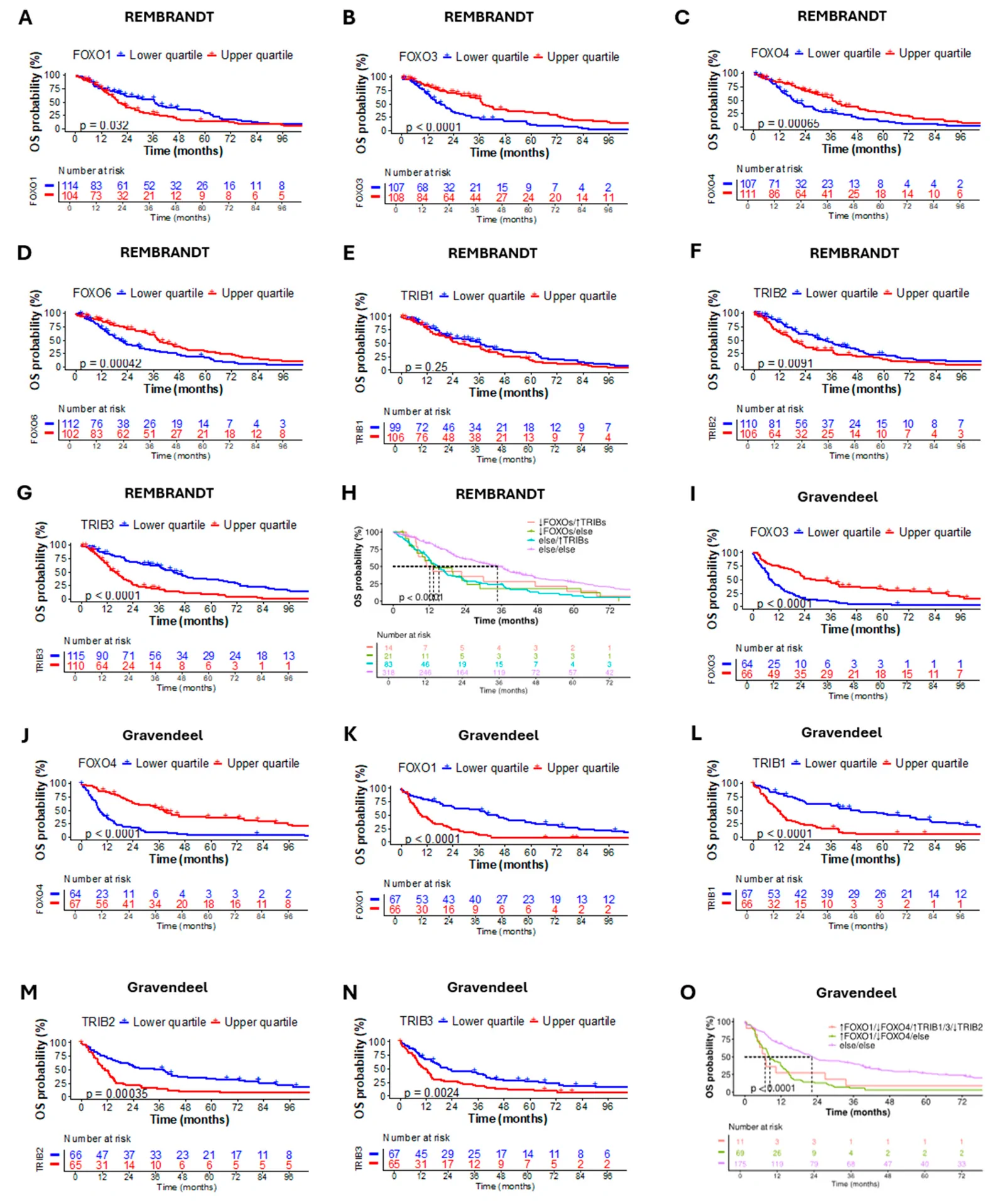
REMBRANDT and Gravendeel microarray datasets validate the mRNA signature and its robustness. (**A**–**G**) Kaplan-Meier plots of FOXO and Tribbles for REMBRANDT glioma samples. The lower quartile, in blue, and the upper quartile, in red, were established as the cut-off for low and high expression levels, respectively. (**A**) FOXO1; (**B**) FOXO3; (**C**) FOXO4; (**D**) FOXO6; (**E**) TRIB1; (**F**) TRIB2; (**G**) TRIB3. All *p*-values refer to log-rank test. (**H**) Kaplan-Meier plot of a combination of low levels of FOXOs plus high levels of Tribbles for REMBRANDT glioma samples. The median value was set as the cut-off. “Else” means every other possible combination of expressions than the stated in comparison. Low FOXO1/3/4/6 mRNA levels combined with high levels of TRIB1/2/3 (↓FOXOs/↑TRIBs) are represented in pink. All *p*-values refer to log-rank test. (**I**–**N**) Kaplan-Meier plots of FOXO and Tribbles for Gravendeel glioma samples. The lower quartile, in blue, and the upper quartile, in red, were established as the cut-off for low and high expression levels, respectively. (**I**) FOXO3; (**J**) FOXO4; (**K**) FOXO1; (**L**) TRIB1; (**M**) TRIB2; (**N**) TRIB3. All *p*-values refer to log-rank test. (**O**) Kaplan-Meier plot of a combination of high levels of FOXO1 and low levels of FOXO4 plus high levels of TRIB1/3 and low levels of TRIB2 for Gravendeel glioma samples. The median value was set as the cut-off. “Else” means every other possible combination of expressions than the stated in comparison High FOXO1 and low FOXO4 mRNA levels combined with high levels of TRIB1/3 and low of TRIB2 (↑FOXO1/↓FOXO4/↑TRIB1/3/↓TRIB2) are represented in pink. All *p*-values refer to log-rank test. Abbreviations: OS, overall survival; FOXO, Forkhead box O; FOXO1, Forkhead box O1; FOXO3, Forkhead box O3; FOXO4, Forkhead box O4; FOXO6, Forkhead box O6; TRIB1, Tribbles homolog 1; TRIB2, Tribbles homolog 2; TRIB3, Tribbles homolog 3; TRIBs, Tribbles.

### 3.3 High Grade Tumors Express ↑FOXO1/↓FOXO3/4/↑TRIB1/2/3 mRNA Levels

Next, we sought to determine whether certain glioma tumor types were overrepresented among those exhibiting a high FOXO1 and high Tribbles expression levels accompanied by low FOXO3/4 expression levels. We found that the ↑FOXO1/↓FOXO3/4/↑TRIBs expression pattern was exclusively observed in patients diagnosed with glioblastoma (GB), or grade 4 astrocytoma (Fig. 3A,B). In fact, the majority of GB cases exhibited either the ↑FOXO1/↓FOXO3/4 or ↑TRIBs profile, or both. This finding suggests that this particular mRNA expression signature is strongly associated with higher-grade gliomas known to be associated with poorer prognosis and diminished overall survival (Fig. S2). Consistently, we hypothesized that the mRNA levels of FOXO1, FOXO3, FOXO4, TRIB1, TRIB2, and TRIB3 varied according to tumor grade. To address this question, we evaluated the mRNA expression levels, from the TCGA database, of each individual FOXO or Tribbles gene between GB and other gliomas. We found that FOXO1 (*p* < 0.0001, Fig. 3C), TRIB1 (*p* < 0.0001, Fig. 3D), TRIB2 (*p* < 0.0001, Fig. 3E) and TRIB3 (*p* < 0.0001, Fig. 3F) are overexpressed in GBs compared to patients with other type of gliomas, whilst FOXO3 (*p* < 0.0001, Fig. 3G) and FOXO4 (*p* < 0.0001, Fig. 3H) mRNA levels are underexpressed.

**Figure 3:**
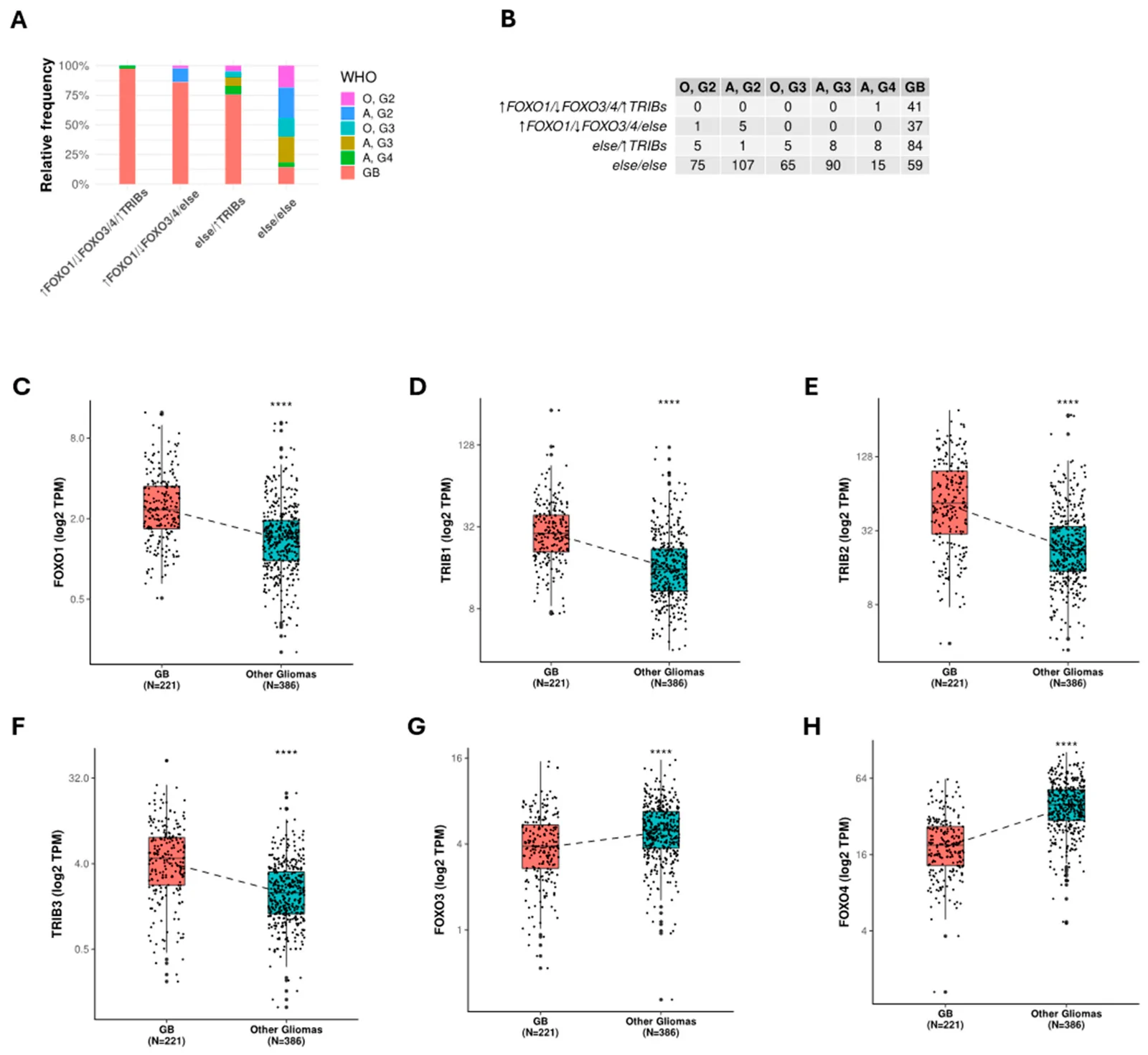
High grade tumors express ↑FOXO1/↓FOXO3/4/↑TRIB1/2/3 mRNA levels. (**A**) Percentage of the different types of tumors for each combination of high levels of FOXO1 and low of FOXO3/4 (↑FOXO1/↓FOXO3/4) and high levels of Tribbles (↑TRIBs) for TCGA glioma samples. “Else” means every other possible combination of expressions than the stated in comparison. (**B**) Number of cases of the different types of tumors for each combination represented in (**A**). (**C**–**H**) Boxplots of FOXO and Tribbles mRNA levels among glioblastoma and other glioma types of TCGA samples. N represents the number of samples in each group. (**C**) FOXO1; (**D**) TRIB1; (**E**) TRIB2; (**F**) TRIB3; (**G**) FOXO3; (**H**) FOXO4. All *p*-values refers to Student’s *t*-test: ****p < 0.0001. Abbreviations: WHO, World Health Organization; G, grade; GB, glioblastoma; TPM, transcripts per million; FOXO, Forkhead box O; FOXO1, Forkhead box O1; FOXO3, Forkhead box O3; FOXO4, Forkhead box O4; TRIB1, Tribbles homolog 1; TRIB2, Tribbles homolog 2; TRIB3, Tribbles homolog 3; TRIBs, Tribbles.

To evaluate the independent prognostic impact of the combined transcriptional profiles of FOXO and Tribbles, we performed a multivariate Cox proportional hazards model, adjusted for established clinical and molecular variables (Fig. 4 and Table S2). The model included age group, gender, IDH mutation status, 1p/19q codeletion, *MGMT* promoter methylation, WHO classification, and FOXO/Tribbles expression profiles. Age was a strong predictor of overall survival, with elderly patients (>64 years) showing a significantly higher risk of death compared with the youngest group (<48 years) (HR = 5.30, 95% CI 2.931–9.6, *p* = 3.5 × 10^−^^8^), while middle-aged patients (48–64 years) had a moderate increase (HR = 1.69, *p* = 0.049). Among molecular markers, wild-type IDH (HR = 3.27, *p* = 0.020) and non-codeleted 1p/19q status (HR = 14.06, *p* < 0.001) were associated with poorer survival, whereas MGMT methylation showed no significant effect (*p* = 0.42). Tumor classification was also prognostically relevant: Oligodendroglioma Grade 3 cases exhibited increased risk compared with Grade 2 (HR = 4.07, *p* = 0.019), while Astrocytoma Grades 2 and 3 were associated with a significantly lower hazard (HR = 0.19, *p* = 0.001; HR = 0.35, *p* = 0.029, respectively). Importantly, the analysis revealed no significant differences in survival between Astrocytoma Grades 4 and GB, showing that they behave similarly in prognostic terms. The FOXO/Tribbles signature did not reach statistical significance in the multivariate model (HR = 1.35, *p* = 0.33), indicating that its prognostic contribution may be influenced by established clinical and molecular variables. Accordingly, the FOXO/Tribbles signature should be interpreted as an associative marker supported by univariate and cross-cohort analyses, rather than an independent prognostic factor.

**Figure 4:**
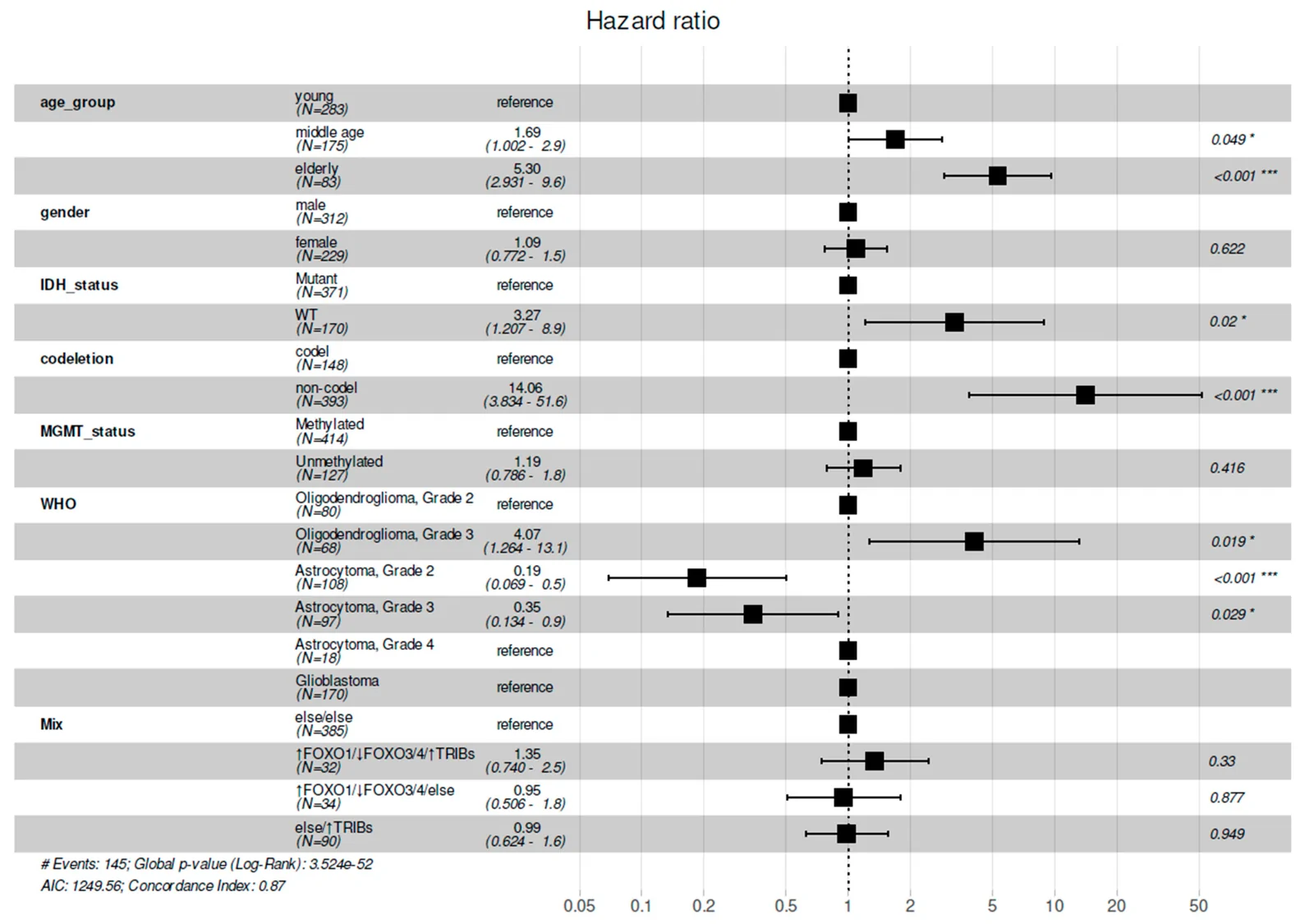
Multivariate Cox proportional hazards regression model assessing potential confounding factors for the combined transcriptional profiles of FOXO and Tribbles. The analysis included age group, gender, *IDH* mutation status, 1p/19q codeletion, *MGMT* promoter methylation, WHO classification, and FOXO/Tribbles expression profiles as covariates. Hazard ratios (HR) with corresponding 95% confidence intervals (CI) and *p*-values were estimated using a Cox proportional hazards model. *p < 0.005, ***p < 0.001. Abbreviations：*IDH*, isocitrate dehydrogenase; *MGMT*, O6-methylguanine-DNA methyltransferase; WHO, World Health Organization.

### 3.4 FOXOs and Tribbles Expression Enable Enhanced Stratification of Grade 4 Glioma Patients

The presence of the IDH mutation in gliomas is a favorable prognostic marker (*p* < 0.0001, Fig. 5A) and targeted therapeutics have been approved to treat patients with grade 2 astrocytoma or oligodendroglioma with mutated IDH [34]. However, patients without mutations in the IDH genes (i.e., patients with GB) are of greater concern as they exhibit worse survival rates and face a lack of effective treatment options. Therefore, it is paramount to find new biomarkers that further stratify these patients and ultimately develop new drugs capable of targeting specific drivers of these tumors. We hypothesized that a signature of high FOXO1, low FOXO3/4, and high TRIB1/2/3 could identify patients likely to benefit from novel therapeutics targeting these families, which are currently in preclinical development [12,35,36]. The majority of patients with high FOXO1 and low FOXO3/4 signature, elevated TRIB1/2/3 levels, or both signatures combined, are IDH wild-type (Fig. 5B). This is unsurprising since these signatures are enriched in GB (Fig. 3A). These findings suggest patients that lack viable treatment options may benefit from evaluating FOXO and Tribbles protein levels as potential therapeutic markers.

Accordingly, we aimed to analyze the robustness of the FOXO/Tribbles signature in stratifying GB patients, as well as grade 4 astrocytomas. Given the aggressive nature of these high-grade tumors both patient groups typically receive similar treatment regimens, making this comparison particularly relevant [37]. We first analyzed the distribution of TCGA patients with grade 4 tumors (astrocytomas or glioblastomas) across different combinations of FOXO and Tribbles signatures. We found that approximately 70% of grade 4 glioma patients express high levels of TRIB1/2/3 and/or decreased FOXO3/4 levels compared to 30% of patients who show no alteration on mRNA levels of both TRIBBLEs and FOXO (Fig. 5C). This means that the majority of grade 4 patients, approximately 55%, would potentially benefit from Tribbles inhibition while 32% could benefit from FOXO modulation. Of these patients eligible for target therapy, 25% would profit from both FOXO and Tribbles therapeutics.

We next compared the discriminatory power of the FOXO/Tribbles signature to that of established glioma biomarkers. A key limitation of the available datasets is the lack of detailed treatment information, particularly on the use and type of chemotherapy. This gap impedes our ability to distinguish whether the observed survival benefits are prognostic or predictive. For instance, while MGMT promoter methylation status is an established predictive biomarker in GB, associated with improved response to temozolomide, its independent prognostic value remains debated [38,39]. Likewise, in astrocytomas *MGMT* promoter is frequently methylated, although its clinical relevance has not yet been as well established as in GB [1]. In order to evaluate the potential clinical relevance of the FOXO/Tribbles signature, we compared its performance to *MGMT* promoter methylation using Kaplan–Meier analysis and calculated the Concordance Index, a metric of the discriminatory ability of a prognostic model (with higher values indicating better discrimination). For the MGMT status, TCGA patients with grade 4 were separated into methylated and unmethylated (*p* = 0.018, Fig. 5D). For the FOXO/Tribbles signature, TCGA patients with grade 4 were separated into patients hypothetically eligible for FOXO and/or Tribbles therapeutics against else/else subgroup of patients (*p* = 0.023, Fig. 5E and Fig. S3).

**Figure 5:**
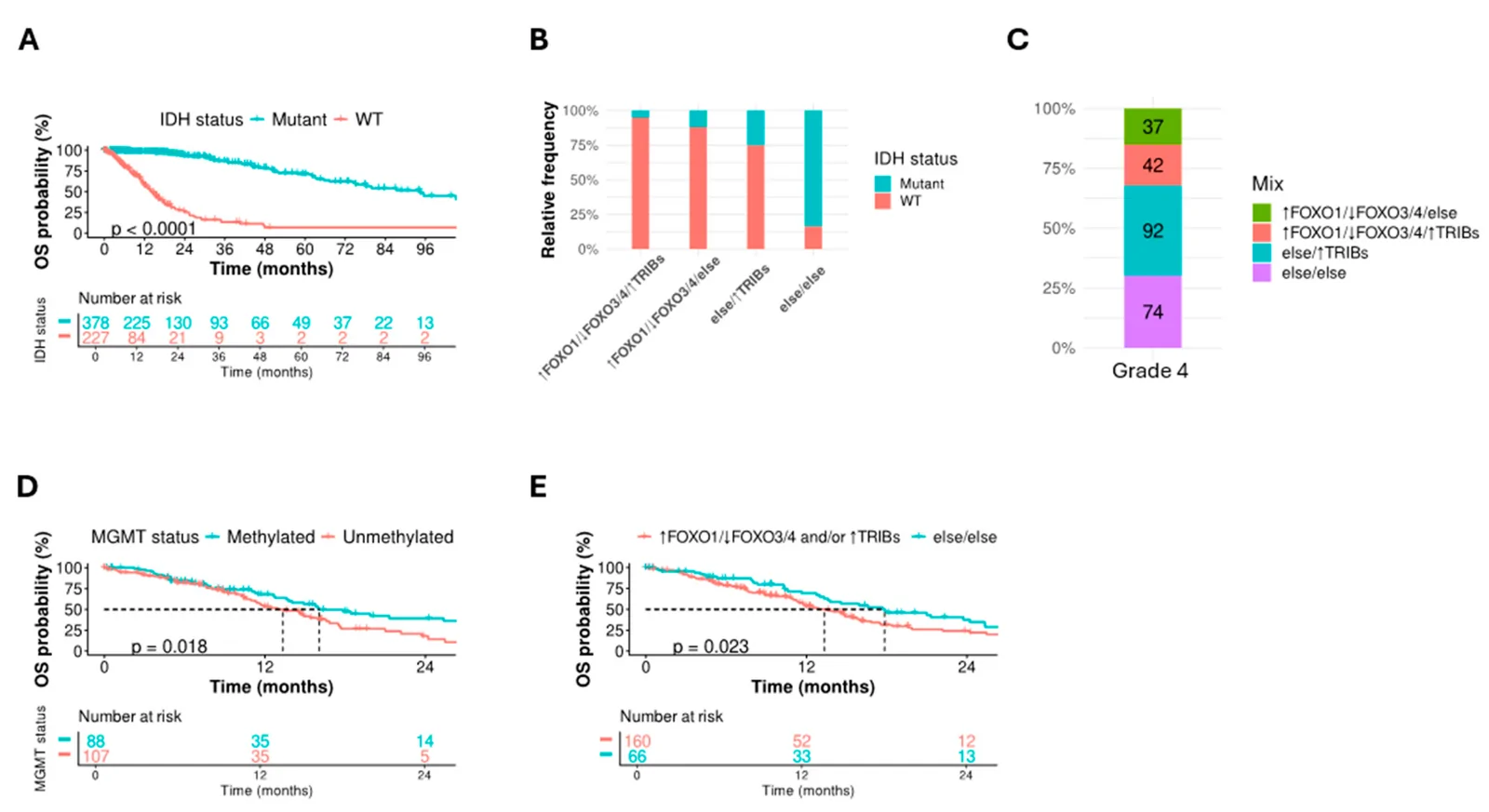
FOXOs and Tribbles expression enable enhanced stratification of grade 4 glioma patients. (**A**) Kaplan-Meier plot of IDH mutation status for the TCGA glioma samples. Mutated in blue and wild type (wt) in pink. (**B**) Percentage of patients with different IDH mutation status for each combination of high levels of FOXO1 and low of FOXO3/4 (↑FOXO1/↓FOXO3/4) and high levels of Tribbles (↑TRIBs) for TCGA glioma samples. “Else” means every other possible combination of expressions than the stated in comparison. (**C**) Number of patients for each combination of high FOXO1 and low FOXO3/4 levels plus high levels of Tribbles for TCGA grade 4 glioma samples. (**D**) Kaplan-Meier plot of MGMT promoter methylation status for the TCGA grade 4 glioma samples. Methylated in blue and unmethylated in pink. (**E**) Kaplan-Meier plot of a combination of FOXOs and Tribbles levels in TCGA grade 4 glioma samples. Subgroups with high FOXO1 and low FOXO3/4 mRNA levels (↑FOXO1/↓FOXO3/4/else), high levels of TRIB1/2/3 (else/↑TRIBs), or both (↑FOXO1/↓FOXO3/4/↑TRIBs) represented together in pink. “Else” means every other possible combination of expressions than the stated in comparison. All *p*-values refer to log-rank test. Abbreviations: IDH, isocitrate dehydrogenase; WT, wild type; MGMT, O-6-methylguanine-DNA methyltransferase; FOXO, Forkhead box O; FOXO1, Forkhead box O1; FOXO3, Forkhead box O3; FOXO4, Forkhead box O4; TRIBs, Tribbles; TRIB1, Tribbles homolog 1; TRIB2, Tribbles homolog 2; TRIB3, Tribbles homolog 3; OS, overall survival.

Our results show that the FOXO/Tribbles signatures offered prognostic discrimination of grade 4 patients in terms of survival beyond epigenetic inhibition of *MGMT*. High FOXO1 and low FOXO3/4 signature, high levels of TRIB1/2/3, or both signatures combined, collectively show a median survival difference of 4.5 months compared to other signature combinations, with a Concordance Index of 0.55. This contrasts with the 2.7-month difference observed for MGMT status (methylated vs. unmethylated), with a Concordance Index of 0.46. These findings suggest that, independently of MGMT status, the FOXO/Tribbles signature has meaningful discriminatory power that may support its utility for patient stratification in the clinical setting. 

Given the controversial roles of FOXO1 [40] and TRIB3 [41] in cancer, and our data supporting their oncogenic functions, we performed experimental validation in GB cells. To this end, we analyzed a panel of GB cells for their expression of TRIB3 and FOXO1 expression. Based on the obtained data we selected U87 cells to silence FOXO1 using RNAi mediated silencing and deplete TRIB3 in U118 cells by using CRISPR/Cas9-mediated knockout. Consistent with our findings in human patient data, Fig. 6 shows that depletion of either TRIB3 or FOXO1 reduced GB cell viability, suggesting that both proteins support survival pathways in GB cells.

**Figure 6:**
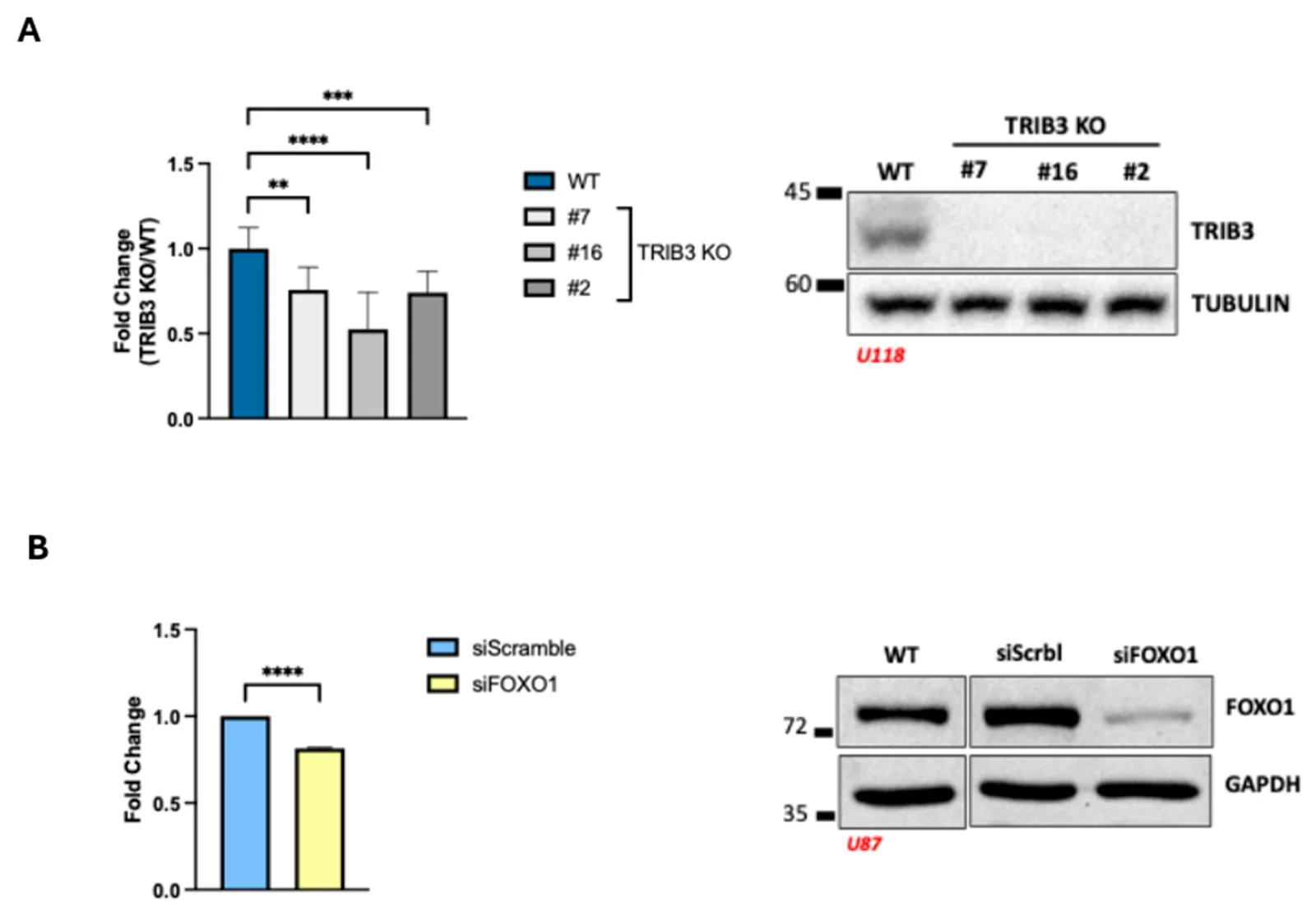
FOXO1 and TRIB3 depletion reduces glioblastoma cell viability. (**A**) Cell viability in U118 wild-type (WT) cells compared with three independent TRIB3 knockout (KO) clones. Immunoblot analysis confirms efficient TRIB3 depletion. Data was analyzed using one-way ANOVA. Significance: **p = 0.0012, ***p = 0.0006, ****p < 0.0001. (**B**) Cell viability in U87 glioblastoma cells transfected with FOXO1-targeting siRNA or control scramble siRNA. Immunoblot verifies downregulation of FOXO1 expression. Data was analyzed using an unpaired *t*-test. Significance. ****p < 0.0001. Data represent mean ± SEM from three independent experiments (n = 3), with each condition assayed in triplicate wells. Together, these data indicate that both TRIB3 and FOXO1 support glioblastoma cell survival. Abbreviations:GAPDH, glyceraldehyde-3-phosphate dehydrogenase; FOXO1, Forkhead box O1; TRIB3, Tribbles homolog 3; β-actin, beta-actin.

Taken together, these results underscore the clinical relevance of the FOXO/Tribbles signature in selecting patients for potential targeted therapies. Notably, 70% of grade 4 gliomas exhibit either a high FOXO1 and low FOXO3/4 signature, elevated TRIB1/2/3 levels, or a combination of both. This highlights the potential of FOXO/Tribbles signatures as an effective tool for guiding treatment strategies.

## 4 Discussion

GB is the most lethal primary brain tumors and are associated with poor response to treatments [2]. Dysregulation of both FOXO [10] and Tribbles [42,43] family members has been implicated in various cancers, including GB, although their precise roles are highly context-dependent [6,44]. In this study, we identified, using the TCGA RNA-seq cohort, a FOXO/Tribbles transcriptional signature associated with glioma survival and subsequently evaluated its biological robustness in three independent transcriptomic cohorts. Although the precise combinations showing the strongest prognostic association varied between datasets, the coordinated dysregulation of FOXO and Tribbles family members consistently identified patients with poorer clinical outcome. Such variability is expected given the differences in transcriptomic platforms (RNA-seq versus microarray), cohort composition [45,46], molecular classification, and analytical methodology [47,48,49]. However, when evaluated in a multivariate Cox proportional hazards model adjusted for established clinical and molecular factors, the combined FOXO/Tribbles signature did not reach statistical significance. This indicates that its prognostic contribution may be partially confounded by variables such as age, IDH status, and 1p/19q codeletion. Therefore, the proposed signature should be interpreted as an associative biomarker supported by univariate and cross-cohort analyses, rather than an independent prognostic factor. 

From a translational perspective, the clinical applicability of the full FOXO/Tribbles signature may be limited, as it identifies a subset of patients rather than the entire glioma population. Furthermore, due to the absence of treatment-stratified data, the signature should be considered prognostic rather than predictive at this stage.

Our results support a tumor suppressor role for FOXO3, FOXO4 and FOXO6, while suggesting that FOXO1 may act as an oncogene in gliomas. Notably, consensus from a vast body of research is that FOXO1 functions primarily as a tumor suppressor. However, highlighting the context-dependent nature of FOXO1, emerging evidence indicates it can also exhibit oncogenic functions. Specifically, FOXO1 was found to promote stemness gene expression in GB models, thereby driving tumor progression. Inhibition of **FOXO1** in GB cellular models induced apoptosis, further suggesting its oncogenic role in GB [50]. Additionally, elevated FOXO1 levels have been associated with upregulation of steroid enzymes facilitating androgen synthesis [51] and increased expression of canonical WNT pathway genes [52], both of which are linked to enhanced tumor proliferation. However, contrasting studies have reported overexpression of FOXO1 has also been associated with increased sensitivity to TMZ, reduced cell adhesion, and impaired migration and invasion abilities of glioma cells [53] and FOXO1 inactivation correlated with significantly shorter overall and progression-free survival [54]. Therefore, we experimentally assessed the impact of FOXO1 silencing in GB cells, showing that it reduces cell viability supporting its oncogenic role.

FOXO3 expression has been reported to be markedly downregulated in glioma tissues compared to adjacent normal tissues and has been associated with transcriptional signatures of neuronal differentiation in certain glioma stem cell lines. It also appears to influence glioma stem cell fate by promoting a shift away from quiescence. Restoration of FOXO3 expression induces cell cycle arrest and inhibits glioma cell proliferation and metastasis [55]. However, paradoxically, FOXO3 has also been shown to induce resistance to TMZ in glioma cells [56]. FOXO4 is consistently reported to be downregulated in GB tissues and cell lines relative to normal brain tissue. Its overexpression significantly inhibits glioma cell proliferation, migration, and invasion, while promoting apoptosis *in vitro*. *In vivo* studies further demonstrate that FOXO4 overexpression suppresses tumor growth [57]. In our analysis, FOXO4 exhibited the strongest association with patient survival, suggesting a predominant tumor suppressor role in glioma. This effect may be attributed to the relatively higher expression of FOXO4 compared to the other isoforms in supportive cells of the human brain (Fig. 3C,G,H). In contrast, FOXO6 displays a more ambiguous functional profile. While its expression is low in adult neural stem cells and not essential for maintaining proliferation, FOXO6 plays a role in mediating cell state transitions, including exit from quiescence and induction of proliferation [58]. Among all FOXO isoforms evaluated in our study, FOXO6 exhibited the weakest discriminatory power between high and low expression groups in survival analysis and contributed minimally to the overall prognostic signature (Fig. S1A). 

Consistent with previous studies [20], our results strongly support the role of the Tribbles proteins TRIB1 and TRIB2 as oncogenic drivers in glioma. Notably, members of the Tribbles family have been widely implicated in mediating therapy resistance across diverse cancer types. TRIB1 expression was not only associated with overall survival in GB patients across multiple cohorts, but its *in vitro* overexpression reduces RT- and TMZ-induced apoptosis by activating pro-survival signaling pathways, including ERK and AKT [59]. Similarly, strong evidence supports a critical role for TRIB2 as a driver of resistance to multiple anticancer therapies across diverse tumor types [15,16,60,61,62,63,64]. A recent study demonstrated that TRIB2 suppresses the transcription of genes involved in antigen presentation and T-cell recruitment by modulating the expression of methylation regulators, thereby promoting an immune-cold tumor microenvironment [65]. TRIB2 has also been implicated in resistance to RT and TMZ in glioma, partly through its association with MAP3K1 expression [42]. The role of TRIB3 in cancer, including gliomas, is complex and points to a highly context-dependent dual activity. Several studies have reported a tumor-suppressor function for TRIB3 [66,67,68]. Notably, Salazar et al. reported that its upregulation is part of a stress response that promotes autophagy in glioma cells after cannabinoid treatment [21]. Conversely, other studies have suggested that TRIB3 functions primarily as an oncogene in various malignancies [69,70,71], including gliomas [72,73]. We provide evidence that TRIB3 may support tumor cell survival in GB models, although its role remains context-dependent.

Taken together, these observations, along with our data, suggest that FOXO1, TRIB1, TRIB2, and TRIB3 may act as drivers of glioma progression, whereas loss of FOXO3, FOXO4, and FOXO6 could contribute to glioma development through tumor-suppressive mechanisms. Therefore, the FOXO and Tribbles families should be considered potential therapeutic targets for this disease, particularly for overcoming therapy resistance and for use in combination with other treatments. Specifically, our data indicate that inhibition of FOXO1 and TRIB1, TRIB2, and TRIB3, alone or in combination and/or activation of FOXO3 and FOXO4 may benefit glioma patients whose tumors exhibit the described signature.

Although non-liganded transcription factors such as FOXO proteins and the pseudokinases of the Tribbles family have historically been considered undruggable [74], recent progress has made targeting these protein families feasible [20,75]. Several small-molecule compounds have been described that interfere with the interaction of FOXO1 with DNA including AS1842856 [76] or specific binding partners [77], leading to FOXO1 inhibition. Conversely, activation of FOXO3 has moved into the spotlight and represents an ideal target for pharmaceutical intervention [12], as FOXO factors are usually not mutated or deleted unlike other tumor suppressors including PTEN or p53, and can be reactivated at multiple levels of regulation, including upstream regulators of post-translational modifications, nuclear export, interactions with binding partners, and stabilization of interactions with consensus DNA-binding sites [78]. Several FDA-approved compounds known to activate FOXO3 are approved for the treatment of various cancer types, including the CRM1 inhibitor Selinexor [79], the mTOR inhibitor everolimus, and multiple receptor tyrosine kinase (RTK) inhibitors [80]. The development of FOXO3 activators remains an active area of research in both academia and industry, and several companies have emerged in recent years to develop therapeutics targeting the FOXO family.

Targeting Tribbles proteins has also emerged as a promising strategy for cancer therapy, with TRIB2 receiving focused attention. Although Tribbles proteins contain a pseudokinase domain, it has been reported that TRIB2 binds ATP and autophosphorylates [81]. Accordingly, a TRIB2 kinase activity assay revealed a novel indazole derivative as a TRIB2-binding molecule [82], which was subsequently used as the warhead for the first TRIB2-PROTAC degrader [35]. Additionally, recent studies have shown that approved EGFR inhibitors, including afatinib, covalently bind and degrade TRIB2 at micromolar concentrations [18], and the antiviral drug daclatasvir can similarly destabilize TRIB2 [83]. We have recently placed data in the public domain demonstrating that several small-molecule compounds most notably CDK12 inhibitors reduce TRIB2 protein levels in cancer cells with hundreds-fold greater potency than afatinib and daclatasvir [84]. Several strategies have also been reported to decrease TRIB3 expression or function, with therapeutic implications for both cancer and metabolic diseases. Peptide-based inhibitors, such as the helix-mimetic peptide PCM4 and the fusion peptide P2-T3A6, disrupt oncogenic protein–protein interactions involving TRIB3, leading to reduced TRIB3 levels and suppression of tumor growth [36]. In addition, several small molecules have been shown to modulate TRIB3 such as the antidiabetic drug metformin [85], as well as the Polo-like kinase inhibitors BI-2536 and volasertib inhibiting *TRIB3* transcription [84]. 

It is important to note that advances in structural biology, together with the use of public databases and emerging tools such as AlphaFold 3, can greatly facilitate the design of selective inhibitors and activators targeting these proteins. Recent studies have used crystal structures of FOXO3 in both its free and DNA-bound states to identify potential binding sites for small molecules capable of either disrupting or stabilizing FOXO3–DNA interaction [86].

Collectively, a variety of strategies are now available to modulate the activity of FOXO/Tribbles protein families. Hypothetically, a therapeutic strategy combining global FOXO activation, global Tribbles inhibition, and FOXO1-specific inhibition could help counteract the transcriptional profile associated with high-grade glioma. However, further studies are required to explore the feasibility, efficacy, and safety of such an approach. In particular, there is no direct evidence regarding the blood-brain barrier (BBB) penetration for most of the compounds discussed previously [87].

We propose to explore the therapeutic potential of FOXO1, FOXO4, TRIB2 and TRIB3 as drug targets to improve the treatment of patients with glioma. Drugs targeting these novel targets, combined with the FOXO/Tribbles signature as companion biomarkers, hold the potential to enhance GB treatment outcomes while sparing patients from ineffective therapies. This suggests that our results can have an impact by establishing new potential therapeutic targets for several cancer types, in particular gliomas. However, the clinical relevance of these observations and the mechanistic of these proteins in gliomas has yet to be fully established. 

A key limitation of this study lies in its correlative nature. While the data establish a consistent relationship between FOXO/Tribbles expression patterns and survival, they do not demonstrate causality or define the mechanistic basis underlying this association. Although FOXO transcription factors are well-established downstream effectors of the PI3K/AKT pathway and Tribbles proteins are known modulators of signaling networks, we do not directly demonstrate that these interactions are functionally operative in glioma. As such, the proposed FOXO–Tribbles axis should be viewed as a biologically plausible hypothesis rather than a validated mechanistic model. A recent study by Liu et al. illustrates how molecular interactions, pathway activation, and functional consequences in GB coalesce into a comprehensive mechanistic loop [88]. 

Furthermore, moving beyond the expression-based prognostic associations requires the integration of multi-omics data profiles to determine pathway-level causality as established by Ubaid et al. [89]. This process can be greatly enhanced by AI-driven, transcriptome-centered multi-omics integration within precision medicine [90]. The functional experiments performed in this study provide limited but supportive evidence. Specifically, depletion of FOXO1 or TRIB3 reduced cell viability in glioblastoma cell lines, suggesting that these proteins may con-tribute to tumor cell survival. However, these findings are restricted to a small number of *in vitro* models and rely on a single assay type. They do not establish generalizability across glioma subtypes, nor do they demonstrate pathway-level effects or downstream mechanisms. Consequently, these results should be interpreted as preliminary and hypothesis-generating. 

Another important consideration is the reliance on bulk transcriptomic datasets. Such datasets are subject to inherent limitations, including cellular heterogeneity, technical variability, and differences in data processing pipelines [91,92]. Collectively, these factors constraint the transition from expression signatures to robust, clinically actionable biomarkers and may also contribute to discrepancies observed across cohorts, including differences in the precise multigene combinations associated with prognosis. These variations are expected given differences in patient populations, transcriptomic platforms, and analytical methodologies, and do not negate the consistent observation that coordinated dysregulation of FOXO and Tribbles family members is associated with poor glioma outcome. Importantly, multigene signatures capture the combined effect of interacting family members; therefore, the prognostic direction of an individual gene should not be expected to remain unchanged when evaluated within the context of the complete transcriptional profile. While the overall pattern remains consistent, these variations highlight the need for cautious interpretation.

Methodologically, the use of quartile- and median-based cutoffs represents an exploratory approach commonly applied in large-scale transcriptomic analyses. However, this strategy does not define clinically actionable thresholds and may introduce bias. Future studies should aim to develop standardized risk models incorporating training and validation cohorts, as well as optimized cutoff values suitable for clinical implementation. Despite dataset-specific variability, a consistent global pattern emerges across all cohorts: whenever statistical significance is observed, low mRNA levels of FOXO3, FOXO4, and FOXO6, and high expression of FOXO1, TRIB1, TRIB2, and TRIB3, are associated with poorer survival outcomes.

## 5 Conclusion

In conclusion, we identify a FOXO/Tribbles transcriptional profile associated with glioma survival across independent datasets. While not independently prognostic, this signature represents a promising candidate for further investigation and may contribute to improved understanding of glioma biology.

## Data Availability

The datasets analyzed in this study are publicly available. TCGA RNA-seq data were obtained from The Cancer Genome Atlas (https://portal.gdc.cancer.gov/) and reclassified according to WHO CNS5 criteria. CGGA RNA-seq datasets were downloaded from the Chinese Glioma Genome Atlas (http://www.cgga.org.cn/). Microarray datasets were retrieved from the Gene Expression Omnibus (GEO) under the accession numbers GSE68848 (REMBRANDT) and GSE16011 (Gravendeel). All additional information required to reanalyze the data in this study will be made available from the lead contact upon request.

## Supplementary Materials

The supplementary material is available online at https://www.techscience.com/doi/10.32604/or.2026.083560/s1.
