# Supplementary material for: Combined Expression of FOXO and Tribbles Proteins Predicts Survival of Glioma Patients
Source: Oncol Res. 2026 Oct 28;34(11):14. doi: 10.32604/or.2026.083560 (PMC13732327; doi:10.32604/or.2026.083560)
Supplement: Supplementary file 1 [file OncolRes-34-83560-s001.zip › TSP_OR_83560-s001.docx]

**Table S1:** Summary of datasets

| **Factor** | **TCGA** | **CGGA** | **REMBRANDT** | **Gravendeel** |
| --- | --- | --- | --- | --- |
| **Total Number** | 705 | 1018 | 552 | 268 |
|  | *Median (LQ, UQ)* | *Median (LQ, UQ)* | Median (LQ, UQ) | Median (LQ, UQ) |
| **Age at diagnosis** (years) | 47.0 (35.0, 59.0) | 42.0 (35.0, 51.0) | - | 51.53 (38.1, 61.4) |
| **Follow-up all cases** (months) | 12.5 (5.3, 23.9) | 27.5 (10.8, 66.7) | 21.9 (10.5, 44.5) | 14.6 (6.7, 39.8) |
| **Follow-up still living** (months) | 12,0 (5.2, 22.6) | 70,9 (42,7, 99,6) | 16.0 (6.0, 31.5) | 69.9 (39.8, 85.8) |
| **Vital status, n (%)** |  |  |  |  |
| **Alive** | 431 (61.1%) | 362 (35.6%) | 87 (15.8%) | 28 (10.4%) |
| **Dead** | 183 (26.0%) | (617 (60.6%) | 349 (63.2%) | 240 (89.6%) |
| **NA** | 91 (12.9%) | 39 (3.83%) | 116 (21.0%) | - |
| **WHO classification, n (%)** |  |  |  |  |
| **Astrocytoma, Grade 2** | 113 (16.0%) | 175 (17.2%) | 61 (11.1%) | 13 (4.9%) |
| **Astrocytoma, Grade 3** | 98 (13.9%) | 214 (21.0%) | 53 (9.6%) | 16 (6.0%) |
| **Astrocytoma, Grade 4** | 24 (3.40%) | - | - | - |
| **Oligodendroglioma, Grade 2** | 81 (11.5%) | 112 (11,0%) | 27 (4.9%) | 8 (3.0%) |
| **Oligodendroglioma, Grade 3** | 70 (9.93%) | 9 (0.9%) | 22 (4.0%) | 44 (16.4%) |
| **Mixed, Grade 2** | - | 94 (9.2%) | 4 (0.7%) | 3 (1.1%) |
| **Mixed, Grade 3** | - | 21 (2.1%) | 3 (0.5%) | 25 (9.3%) |
| **Glioblastoma, Grade 4** | 221 (31.3%) | 388 (38.2%) | 190 (34.4%) | 159 (59.3%) |
| **NA** | 98 (13.9%) | 5 (0.5%) | 192 (34.8%) | - |
| **IDH status, n (%)** |  |  |  |  |
| **wt** | 237 (33.6%) | 435 (42.7%) | - | - |
| **mutant** | 430 (61.0%) | 531 (52.2%) | - | - |
| **NA** | 38 (5.4%) | 52 (5.1%) | - | - |
| **1p19q codeletion status, n (%)** |  |  |  |  |
| **non-codel** | 498 (70.6%) | 728 (71.5%) | - | - |
| **codel** | 169 (24.0%) | 212 (20.8%) | - | - |
| **NA** | 38 (5.4%) | 78 (7.7%) | - | - |
| **MGMT status, n (%)** |  |  |  |  |
| **Methylated** | 477 (67.7%) | 472 (46.4%) | - | - |
| **Unmethylated** | 164 (23.3%) | 376 (36.9%) | - | - |
| **NA** | 64 (9.1%) | 170 (16.7%) | - | - |

Abbreviations: TCGA, The Cancer Genome Atlas; CGGA, Chinese Glioma Genome Atlas; REMBRANDT, Repository for Molecular Brain Neoplasia Data; WHO, World Health Organization; IDH, Isocitrate Dehydrogenase; 1p19q, chromosome 1p/19q codeletion; MGMT, O-6-Methylguanine-DNA Methyltransferase; NA, not available; LQ, lower quartile; UQ, upper quartile.

**Table S2:** Hazard Ratio

| **Variables** | **Hazard Ratio** | ***p*** | **95% Confidence Interval** | |
| --- | --- | --- | --- | --- |
|  |  |  | **Lower Bound** | **Upper Bound** |
| **Age** (v. young, <48) |  |  |  |  |
| middle age (48-64) | 1.690 | 0.049035 | 1.002 | 2.900 |
| elderly (>64) | 5.304 | 3.54E-08 | 2.931 | 9.600 |
| **Gender** (v. male) |  |  |  |  |
| female | 1.091 | 0.621516 | 0.772 | 1.500 |
| **IDH status** (v. mutated) |  |  |  |  |
| wt | 3.273 | 0.019807 | 1.207 | 8.900 |
| **1p19q status** (v. codel) |  |  |  |  |
| non-codel | 14.061 | 0.000067 | 3.834 | 51.600 |
| **MGMT status** (v. methylated) |  |  |  |  |
| Unmethylated | 1.186 | 0.415519 | 0.786 | 1.800 |
| **WHO** (v. Oligodendroglioma, Grade 2) |  |  |  |  |
| Oligodendroglioma, Grade 3 | 4.072 | 0.018676 | 1.264 | 13.100 |
| Astrocytoma, Grade 2 | 0.186 | 0.000883 | 0.069 | 0.500 |
| Astrocytoma, Grade 3 | 0.347 | 0.029491 | 0.134 | 0.900 |
| Astrocytoma, Grade 4 | NA | NA |  |  |
| Glioblastoma | NA | NA |  |  |
| **FOXO/Tribbles status** (v. else/else) |  |  |  |  |
| ↑FOXO1/↓FOXO3/4/↑TRIBs | 1.348 | 0.32957 | 0.740 | 2.500 |
| ↑FOXO1/↓FOXO3/4/else | 0.951 | 0.876803 | 0.506 | 1.800 |
| else/↑TRIBs | 0.985 | 0.948892 | 0.624 | 1.600 |

Abbreviations: IDH, Isocitrate Dehydrogenase; 1p19q, chromosome 1p/19q codeletion; MGMT, O-6-Methylguanine-DNA Methyltransferase; WHO, World Health Organization; FOXO, Forkhead Box O; TRIBs, Tribbles; NA, not available.

**Supplementary Figure S1:** High FOXO1, low FOXO3/FOXO4, and elevated TRIB1/2/3 mRNA levels form a molecular signature predictive of clinical outcomes. (**A**) Kaplan-Meier plot of all possible combinations of low and high levels of FOXOs for TCGA glioma samples. Median value was set as the cut-off Profiles with worse survival are highlighted in red. (**B-E**) Kaplan-Meier plots of FOXO for CGGA glioma samples. The lower quartile, in blue, and the upper quartile, in red, were established as the cut-off for low and high expression levels, respectively. (**B**) FOXO1; (**C**) FOXO4; (**D**) FOXO3; (**E**) FOXO6. (**F**) Kaplan-Meier plot of all possible combinations of low and high levels of Tribbles levels for TCGA glioma samples. Median value was set as the cut-off. Profile with worse survival is highlighted in red. (**G-I**) Kaplan-Meier plots of Tribbles for CGGA glioma samples. The lower quartile, in blue, and the upper quartile, in red, were established as the cut-off for low and high expression levels, respectively. (**G**) TRIB1; (**H**) TRIB2; (**I**) TRIB3. (**J**) Kaplan-Meier plot of a combination of high levels of FOXO1 and low levels of FOXO3/4 plus high levels of Tribbles for CGGA glioma samples. The median value was set as the cut-off. “Else” means every other possible combination of expressions than the stated in comparison. High FOXO1 and low FOXO3/4 mRNA levels combined with high levels of TRIB1/2/3 (↑FOXO1/↓FOXO3/4/↑TRIBs) are represented in pink. All p-values refer to log-rank test. Abbreviations: TCGA, The Cancer Genome Atlas; CGGA, Chinese Glioma Genome Atlas; FOXO, Forkhead box O; FOXO1, Forkhead box O1; FOXO3, Forkhead box O3; FOXO4, Forkhead box O4; FOXO6, Forkhead box O6; TRIBs, Tribbles; TRIB1, Tribbles homolog 1; TRIB2, Tribbles homolog 2; TRIB3, Tribbles homolog 3; GB, glioblastoma; mRNA, messenger RNA;OS, overall survival .

**Supplementary Figure S2:** Kaplan-Meier plot of the different types of tumors for TCGA glioma samples. p-value refers to log-rank test. Abbreviations: TCGA, The Cancer Genome Atlas; GB, glioblastoma; OS,Overall Survival;WHO, World Health Organization.

**Supplementary Figure S3:** (**A**) Kaplan-Meier plot of a combination of FOXOs and Tribbles levels in TCGA GB samples. (**B**) Kaplan-Meier plot of a combination of FOXOs and Tribbles levels in TCGA grade 4 astrocytoma samples. Subgroups with high FOXO1 and low FOXO3/4 mRNA levels (↑FOXO1/↓FOXO3/4/else), high levels of TRIB1/2/3 (else/↑TRIBs), or both (↑FOXO1/↓FOXO3/4/↑TRIBs) represented together in pink. “Else” means every other possible combination of expressions than the stated in comparison. All p-values refer to log-rank test. Abbreviations: TCGA, The Cancer Genome Atlas; GB, glioblastoma; FOXO, Forkhead box O; FOXO1, Forkhead box O1; FOXO3, Forkhead box O3; FOXO4, Forkhead box O4; TRIBs, Tribbles; TRIB1, Tribbles homolog 1; TRIB2, Tribbles homolog 2; TRIB3, Tribbles homolog 3; mRNA, messenger RNA.
